# StaRTrEK:*in silico* estimation of RNA half-lives from genome-wide time-course experiments without transcriptional inhibition

**DOI:** 10.1186/s12859-022-04730-x

**Published:** 2022-05-20

**Authors:** Federica Conte, Federico Papa, Paola Paci, Lorenzo Farina

**Affiliations:** 1 Institute for Systems Analysis and Computer Science “Antonio Ruberti”, National Research Council, Rome, Italy; 2 Department of Computer, Control and Management Engineering, Sapienza University of Rome, Rome, Italy; 3 SysBio Centre for Systems Biology, Milan, Italy

**Keywords:** RNA half-lives, Gene expression time profiles, Computational biology, Bioinformatics

## Abstract

**Background:**

Gene expression is the result of the balance between transcription and degradation. Recent experimental findings have shown fine and specific regulation of RNA degradation and the presence of various molecular machinery purposely devoted to this task, such as RNA binding proteins, non-coding RNAs, etc. A biological process can be studied by measuring time-courses of RNA abundance in response of internal and/or external stimuli, using recent technologies, such as the microarrays or the Next Generation Sequencing devices. Unfortunately, the picture provided by looking only at the transcriptome abundance may not gain insight into its dynamic regulation. By contrast, independent simultaneous measurement of RNA expression and half-lives could provide such valuable additional insight. A computational approach to the estimation of RNAs half-lives from RNA expression time profiles data, can be a low-cost alternative to its experimental measurement which may be also affected by various artifacts.

**Results:**

Here we present a computational methodology, called StaRTrEK (STAbility Rates ThRough Expression Kinetics), able to estimate half-life values basing only on genome-wide gene expression time series without transcriptional inhibition. The StaRTrEK algorithm makes use of a simple first order kinetic model and of a $l_1$-norm regularized least square optimization approach to find its parameter values. Estimates provided by StaRTrEK are validated using simulated data and three independent experimental datasets of two short (6 samples) and one long (48 samples) time-courses.

**Conclusions:**

We believe that our algorithm can be used as a fast valuable computational complement to time-course experimental gene expression studies by adding a relevant kinetic property, i.e. the RNA half-life, with a strong biological interpretation, thus providing a dynamic picture of what is going in a cell during the biological process under study.

**Supplementary Information:**

The online version contains supplementary material available at 10.1186/s12859-022-04730-x.

## Introduction

Transcript levels are tightly regulated by many and coordinated molecular machinery to obtain the proper balance between RNA production and degradation. In the past, focus of the research has been on transcriptional regulation and on the resulting regulatory network [7, 16]. By contrast, experimental findings have shown that fine and specific regulation of degradation is needed to a proper orchestration of cell response to internal/external stimuli [9, 10, 19]. It is now widely recognized that transcript degradation is not a simple ‘disposal system’ but it is an essential post-transcriptional regulatory layer acting in all organisms and playing an important role in determining the proper levels of gene products [1, 9]. Regulation of transcripts’ stability (*a.k.a.* half-lives), often mediated by specific proteins and non-coding RNAs, is emerging as a key regulator of gene expression, impacting development, cell fate and much more [1].

Since stability control is both transcript-specific and process-specific [6], to understand the complexity of gene regulatory networks, it is necessary to complement usual time-course experiments, with decay rates measurements in the same condition [5, 7]. However, the simultaneous measurement of both half-lives and time-courses can be very expensive and RNA stability measurement protocol may significantly affect cellular physiology [12, 18]. Therefore, an alternative computational approach that make use of time-courses only, can be very useful to provide further insight into the dynamics of the biological process under study.

Here we developed an *in silico* methodology called StaRTrEK (STAbility Rates ThRough Expression Kinetics), able to reliably estimate half-lives from short time series without transcriptional inhibition, i.e., from at least 5–6 time points. The latter feature of the algorithm is very important in time-course experiments, since in most cases only few samples are experimentally measured over time. By contrast, for example, in physiological modeling [17] the measurements’ sampling time can be very high since the number of samples do not affect significantly experimental costs.

**Fig. 1:**
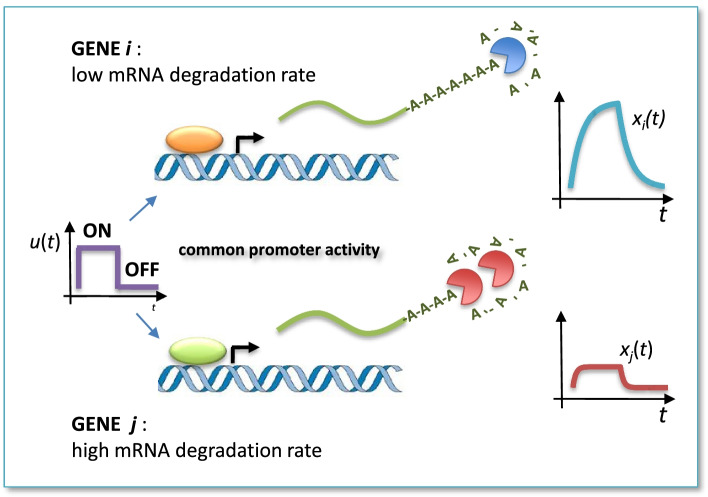
Illustration of the mathematical model underlying StaRTrEK. Gene pairs used to estimated half-lives are assumed to have a common (up to a scaling factor) promoter activity and different half-life values that can explain the different shape of the gene expression time profile

StaRTrEK relies on a computational model of post-transcriptional regulation based on first order kinetics and a least square optimization with $l_1$-norm regularization approach for robust parameters’ estimation. Notwithstanding its simplicity, the proposed algorithm is able to explain the observed differences in RNA levels’ dynamics as a consequence of different decay rates in the presence of a common—up to a scaling factor—promoter activity. The ability of the proposed algorithm to recover half-lives from short time series is based on its simplicity, i.e., on the small number of model parameters to be estimated (just three), as opposed to more sophisticated but complicated models requiring long time series for reliable estimates of many parameters [4]. Precisely, the model assumes that, when a pair of genes have a correlated RNA production rate but different shape of the gene expression time profile, the latter can be ascribed to differences in their stability rate and not to different promoter activities. This key point is illustrated by Fig. 1.

To prove the validity of the proposed methodology, we preliminary tested the algorithm performances on several types of artificial data, varying the number of samples, as well the type and amplitude of measurement noise. Most importantly, we tested the methodology on real experimental data by comparing StaRTrEK predictions versus two recent public datasets composed of simultaneous measurements of a short genome-wide gene expression time-courses (6 samples) and the corresponding transcripts’ half-lives. We found a highly significant agreement between estimated and measured stability rates. Finally, the algorithm has been also tested on experimental measures of half-lives and *long* gene expression time-series (48 time points), providing an excellent significant agreement between StaRTrEK predictions and measured values.

In conclusion, we believe that our algorithm can be used as a fast valuable computational complement to time-course experimental studies by adding a relevant kinetic property with a strong biological interpretation.

## Method

The balance between transcription and degradation resulting in an appropriate RNA level can be effectively represented using first-order kinetics [12, 13]. Given a set of *m* gene expression time profiles, the rate of change of RNA concentration for two genes, say gene *i* and gene *j*, can be described by:

1 $$\begin{aligned} \left\{ \begin{aligned} \frac{dx_{i}(t)}{dt}&= P_i(t)- k_i x_i(t),\\ \frac{dx_{j}(t)}{dt}&= P_j(t)- k_j x_j(t), \end{aligned} \right. \end{aligned}$$

where $x_i(t)$ and $x_j(t)$ are the measured RNA time profiles of the genes *i* and *j*, $P_i(t)>0$ and $P_j(t)>0$ are their corresponding promoters’ activities, and $k_i>0$ and $k_j>0$ are the specific transcript degradation rates for gene *i* and *j*, respectively. Note that, in usual gene expression experiments, promoter activity *P*(*t*) is not measured and therefore equations (1) cannot be used directly for degradations rates *k* estimate.

Among all $m(m-1)/2$ possible pairs described by equation (1), StaRTrEK algorithm selects those having specific features that makes them ideal candidates for reliable estimation of decay rates $k_i$ and $k_j$ without the use of *P*(*t*). In fact, since such promoter activities are not measured, we want to select, among all available gene pairs, those having the same (up to a scaling factor) promoter activity as illustrated by Fig. 1.

This amounts to saying that we are searching for gene pairs (i, j) having the following property:

2 $$\begin{aligned} \begin{aligned} P_i(t)&= \gamma _i u(t)>0,\\ P_j(t)&= \gamma _j u(t) >0, \end{aligned} \end{aligned}$$

where *u*(*t*) is the unknown common promoter activity function, and $\gamma _i>0$, $\gamma _j>0$ are unknown positive scaling factors. Note that, by means of this procedure, we can obtain a single equation where promoter activities are not used. In fact, substituting equations (2) into (1), we have:

3 $$\begin{aligned} \left\{ \begin{aligned} \frac{dx_{i}(t)}{dt}&= \gamma _i u(t)- k_i x_i(t),\\ \frac{dx_{j}(t)}{dt}&= \gamma _j u(t)- k_j x_j(t). \end{aligned} \right. \end{aligned}$$

Then, obtaining *u*(*t*) from the first equation and by substituting it into the second equation of system (3), we finally obtain:

4 $$\begin{aligned} \frac{dx_j(t)}{dt} = \frac{\gamma _j}{\gamma _i} \frac{dx_i(t)}{dt} + \frac{\gamma _j}{\gamma _i} k_i x_i(t) -k_j x_j(t). \end{aligned}$$

It is worth noting that the only unknown parameters to be estimated in (4) from experimental data are the three values contained in vector $\theta _R$:

$$\begin{aligned} \theta _R^T = \left[ \frac{\gamma _j}{\gamma _i}, \frac{\gamma _j}{\gamma _i} k_i, k_j \right] = \left[ \theta _{1,R}\,\,\, \theta _{2,R}\,\,\, \theta _{3,R}\right] . \end{aligned}$$

where, notably, the unknown term *u*(*t*) is absent. Again, it is important to realize that only three parameters have to be estimated from each gene expression time series.

Equation (4) cannot be directly used for a reliable parameters estimation for many reasons. Firstly, equation (4) contains time derivatives, and their computation from noisy data is notoriously unreliable. Secondly, the functions in equation (4) are continuous in *t*, whilst data measurements are available only at (often few) discrete time points. Then, equation (4) has been discretized. To reduce noise, we preliminary performed a trapezoidal integration of expression (4), obtaining the following discrete-time equation for each of the *n* time samples $\delta$:

5 $$\begin{aligned} D_j^{\delta } = \frac{\gamma _j}{\gamma _i} D_i^{\delta } + \frac{\gamma _j}{\gamma _i} k_i I_i^{\delta }-k_j I_j^{\delta }, \;\;\;\;\;\;\;\; \delta =1,\ldots ,n, \end{aligned}$$

where

$$\begin{aligned} D_i^{\delta }&= x_i(t_{\delta +1}) - x_i(t_{\delta }), \\ D_j^{\delta }&= x_j(t_{\delta +1}) - x_j(t_{\delta }), \\ I_i^{\delta }&= \int _{t_{\delta }}^{t_{\delta +1}} x_i(t) dt \simeq \left[ \frac{x_i(t_{\delta +1}) + x_i(t_{\delta })}{2} \right] (t_{\delta +1} - t_{\delta }), \\ I_j^{\delta }&= \int _{t_{\delta }}^{t_{\delta +1}} x_j(t) dt \simeq \left[ \frac{x_j(t_{\delta +1}) + x_j(t_{\delta })}{2} \right] (t_{\delta +1} - t_{\delta }). \end{aligned}$$

By considering equation (5)—that we called *backward (or reverse) equation*—for each time sample, we obtained a set of linear equations that can be compactly written in matricial form:

$$\begin{aligned} (D_j^1, \ldots , D_j^n) = \left( \frac{\gamma _j}{\gamma _i}, \frac{\gamma _j}{\gamma _i} k_i, k_j \right) \left( \begin{array}{ccccc} D_i^1 &{} \ldots &{} D_i^{\delta } &{} \ldots &{} D_i^n \\ I_i^1 &{} \ldots &{} I_i^{\delta } &{} \ldots &{} I_i^n \\ -I_j^1 &{} \ldots &{} -I_j^{\delta } &{} \ldots &{} -I_j^n \end{array} \right) . \end{aligned}$$

It is worth noting that the time samples may not be necessary taken at equally spaced time intervals, which is actually often the case in biological time-courses experiments. Moreover, by defining the following matrices

$$\begin{aligned} A_R^T = \left( \begin{array}{ccccc} D_i^1 &{} \ldots &{} D_i^{\delta } &{} \ldots &{} D_i^n \\ I_i^1 &{} \ldots &{} I_i^{\delta } &{} \ldots &{} I_i^n \\ -I_j^1 &{} \ldots &{} -I_j^{\delta } &{} \ldots &{} -I_j^n \end{array} \right) \end{aligned}$$

and

$$\begin{aligned} B_R^T = (D_j^1, \ldots , D_j^n), \end{aligned}$$

we can rewrite equations (5) as follows:

6 $$\begin{aligned} A_R\theta _R = B_R, \end{aligned}$$

Analogously, by deriving *u*(*t*) from the second equation of (3) and by substituting it into the first equation, we finally arrived to the following:

7 $$\begin{aligned} \frac{dx_i(t)}{dt} = \frac{\gamma _i}{\gamma _j} \frac{dx_j(t)}{dt} + \frac{\gamma _i}{\gamma _j} k_j x_j(t) -k_i x_i(t), \end{aligned}$$

where the only unknowns are the three parameters vector $\theta _F$:

$$\begin{aligned} \theta _F^T = \left[ \frac{\gamma _i}{\gamma _j}, \frac{\gamma _i}{\gamma _j} k_j, k_i \right] = \left[ \theta _{1,F}\,\,\, \theta _{2,F}\,\,\, \theta _{3,F}\right] . \end{aligned}$$

As for the backward case, we performed an integration of equation (7) and obtained for each time sample $\delta$ the following expression, that we called *forward (or direct) equation*:

8 $$\begin{aligned} D_i^{\delta } = \frac{\gamma _i}{\gamma _j} D_j^{\delta } + \frac{\gamma _i}{\gamma _j} k_j I_j^{\delta }-k_i I_i^{\delta }, \;\;\;\;\;\;\;\; \delta =1,\ldots ,n. \end{aligned}$$

By considering the forward equation for each time sample, we obtained the matricial form:

$$\begin{aligned} (D_i^1, \ldots , D_i^n) = \left( \frac{\gamma _i}{\gamma _j}, \frac{\gamma _i}{\gamma _j} k_j, k_i \right) \left( \begin{array}{ccccc} D_j^1 &{} \ldots &{} D_j^{\delta } &{} \ldots &{} D_j^n \\ I_j^1 &{} \ldots &{} I_j^{\delta } &{} \ldots &{} I_j^n \\ -I_i^1 &{} \ldots &{} -I_i^{\delta } &{} \ldots &{} -I_i^n \end{array} \right) . \end{aligned}$$

Moreover, defining

$$\begin{aligned} A_F^T = \left( \begin{array}{ccccc} D_j^1 &{} \ldots &{} D_j^{\delta } &{} \ldots &{} D_j^n \\ I_j^1 &{} \ldots &{} I_j^{\delta } &{} \ldots &{} I_j^n \\ -I_i^1 &{} \ldots &{} -I_i^{\delta } &{} \ldots &{} -I_i^n \end{array} \right) \end{aligned}$$

and

$$\begin{aligned} B_F^T = (D_i^1, \ldots , D_i^n), \end{aligned}$$

we can write equations (8) as

9 $$\begin{aligned} A_F\theta _F = B_F. \end{aligned}$$

Summarizing, for each pairs of genes *i* and *j*, we have to solve the backward and the forward equations

$$\begin{aligned} \left\{ \begin{array}{ccccl} A_R\theta _R &{} = &{} B_R, &{} &{} \text {backward equation}, \\ A_F\theta _F &{} = &{} B_F, &{} &{} \text {forward equation}, \end{array} \right. \end{aligned}$$

i.e. we have to find the unknown parameters $\theta _R$ and $\theta _F$ from the given data matrices $A_R$ and $A_F$. Notwithstanding the similarities between equation (5) and (8), solutions may be different. In fact, biological data are heavily affected by noise. Thus, the performance of the optimization algorithm used to solve equation (6) or (9), may be quite different.

Moreover, equations (6) and (9), can be written as:

10 $$\begin{aligned} y=A\theta , \end{aligned}$$

where $y=B_R$, $A=A_R$ and $\theta =\theta _R$ for the backward equation case or $y=B_F$, $A=A_F$ and $\theta =\theta _F$ for the forward equation case. Since there are more time samples then parameters (i.e., $n>3$) and matrix *A* is full row rank, we have to choose the solution $\theta$ by minimizing an appropriate cost function. It is known that biological data are affected by noise and that the number of available time samples is usually not much larger the number of parameters to be estimated (three, in our case). Therefore, a typical least squares solution may lead to over-fit, i.e. the situation in which a small mean square error (MSE) may not guarantee quality of estimations. Consequently, we selected an appropriate cost function by following the approach proposed by Kim and co-workers [11], that considered a regularized re-formulation of a standard least squares estimation by defining the following optimization problem:

11 $$\begin{aligned} \text {argmin} \,\,\, ||A\theta -y||_2^2 + \alpha ||\theta ||_1, \end{aligned}$$

where $\alpha$ is a positive scalar coefficient, $\theta$ is a positive parameter vector $\theta \in {\mathbb {R}}^3$, $y \in {\mathbb {R}}^n$ and $A \in {\mathbb {R}}^{n \times 3}$, with a number of available samples $n>3$. Note that $||\xi ||_2$ and $||\xi ||_1$ denote the $l_2$ ($(\sum _i \xi _i^2)^{1/2}$) and, respectively, the $l_1$ ($\sum _i |\xi _i|$) norms of the vector $\xi$.

Problem (11) is called $l_1$-regularized least squares problems (LSPs) and, besides preventing over-fitting, it is also used for signal recovery in the presence of noise. Moreover, we must ensure non-negativity of the parameter vector $\theta$ for each pair of gene expression time-courses, so we finally obtained:

12 $$\begin{aligned} \begin{array}{ll} \text {argmin} \,\,\, ||A\theta -y||_2^2 + \alpha \sum _{i=1}^3 \theta _i, &{}\\ \,\,\,\,\, \theta &{} \\ \text {s.t.} \,\,\,\,\,\,\,\,\,\,\,\,\, \theta _i \ge 0, \;\;\;\;\;\;\;\;\;\;\;\; i=1,2,3. &{} \end{array} \end{aligned}$$

By solving the two optimization problems (12), derived from equations (6), (9) for a given gene pair (*i*, *j*), we obtained two half-life estimates ($h_{iR}, h_{jF}$), related to the solutions $\theta _R$ and $\theta _F$ (backward and forward) as follows:

13 $$\begin{aligned} h_{i,R}= & {} \ln 2/ k_i = \ln 2\cdot \frac{\theta _{1,R}}{\theta _{2,R}}, \end{aligned}$$

14 $$\begin{aligned} h_{j,F}= & {} \ln 2 / k_j = \ln 2\cdot \frac{ \theta _{1,F}}{\theta _{2,F}}. \end{aligned}$$

It is worth noting that the total number of optimization problems to be solved is $m(m-1)$ (two for any pairs among the $m(m-1)/2$ combinations of genes) but they can be computed independently of one another, i.e., in parallel, thus saving computing time.

Let us now collect the total $m(m-1)$ estimates of the *m* unknown half-lifes and the related fit errors ($q=||A\theta -y||_2^2$) into the following square matrices of ${\mathbb {R}}^{m\times m}$:

15 $$\begin{aligned} H=\left( \begin{array}{ccccc} 0 &{} h_{1,R}^{1,2} &{} h_{1,R}^{1,3} &{} \ldots &{} h_{1,R}^{1,m} \\ \\ h_{2,F}^{1,2} &{} 0 &{} h_{2,R}^{2,3} &{} \ldots &{} h_{2,R}^{2,m} \\ \\ h_{3,F}^{1,3} &{} h_{3,F}^{2,3} &{} 0 &{} \ldots &{} h_{3,R}^{3,m} \\ \\ \ldots &{} \ldots &{} \ldots &{} \ldots &{} \ldots \\ \\ h_{m,F}^{1,m} &{} h_{m,F}^{2,m} &{} \ldots &{} h_{m,F}^{m-1,m} &{} 0 \end{array} \right) , \quad Q=\left( \begin{array}{ccccc} 0 &{} q_{1,R}^{1,2} &{} q_{1,R}^{1,3} &{} \ldots &{} q_{1,R}^{1,m} \\ \\ q_{2,F}^{1,2} &{} 0 &{} q_{2,R}^{2,3} &{} \ldots &{} q_{2,R}^{2,m} \\ \\ q_{3,F}^{1,3} &{} q_{3,F}^{2,3} &{} 0 &{} \ldots &{} q_{3,R}^{3,m} \\ \\ \ldots &{} \ldots &{} \ldots &{} \ldots &{} \ldots \\ \\ q_{m,F}^{1,m} &{} q_{m,F}^{2,m} &{} \ldots &{} q_{m,F}^{m-1,m} &{} 0 \end{array} \right) . \end{aligned}$$

Note that we placed the half-life estimates of the type (13) and (14) into the matrix *H* at symmetrical entries w.r.t. the principal diagonal, that is on row *i*—column *j*—and, respectively, on row *j*—column *i*, while the related fit errors into the matrix *Q* following the same placement criterion. Row *i* of matrix *H* contains the $m-1$ estimates that result from all gene pairs (*i*, *j*) with $i \ne j$ and the corresponding errors are contained in the same row of matrix *Q*. The next step is therefore the appropriate selection of the *best* estimates (i.e., the finding of an error threshold) and their integration (averaging) in a single value.

To summarize the algorithm steps, we can identify four phases for the application of StaRTrEK: pre-processing, optimization, filtering and averaging. The pre-processing phase requires gene expression time profiles normalization (zero mean, unit standard deviation), which may be followed by a sampling regularization procedure. In fact, a non uniform time sampling may lead to an ill-conditioned data matrix *A* in the presence of a large variability of the integral values $I^{\delta }_{i}$. To overcome this problem, in case of non-uniform sampling and before using the trapezoidal rule to compute integrals, we suggest to divide time-series into the smallest number of trapezoids with comparable areas. In fact, by doing so, the data entries in matrix *A* will have similar magnitude. The choice of the regularization parameter $\alpha$ in (12) has been done using the so-called “L-curve” method [8]. Once the optimal $\alpha$ parameter has been selected in the previous step, the optimization phase can take place and consists in the computation of the parameter vector $\theta$ by solving (12) for each of the $m(m-1)$ optimization problems (backward and forward problems related to the $m(m-1)/2$ possible gene pairs) and, consequently, in the derivation of corresponding error *q*. The output of the optimization step is therefore the matrix pair *H* and *Q*.

The filtering phase consists of removing from matrix *H* those entries corresponding to large MSEs value by selecting a maximal error threshold $q_{\text {max}}$. The choice of $q_{\text {max}}$ must take into account both the need for a small error and that of a large enough value to have a sufficiently large pool of estimations for reliable half-life averaging (last step of the algorithm). To find an objective way to select the $q_{\text {max}}$ value, we computed the *p* value of a Kolmogorov-Smirnov test between the actual distribution of estimated half-lives and those obtained after a random permutation (shuffling) of time samples for each gene. More precisely, for each pair of time-series, we randomly shuffling one of them, thus mimicking the situation in which one of the two time series is purely random. *p* Values were corrected for multiple testing by computing a false discovery rate (FDR) using the Benjamini-Hochberg procedure [20] and a threshold was set at $FDR<0.05$. The $q_{\text {max}}$ value was selected as the smallest percentile of the MSE distribution able to guarantee, at least, that the 90$\%$ of estimated half-lives is such that $FDR<0.05$. This choice for $q_{\text {max}}$ allows to maximize the total number of genes with a reliable half-life evaluation, while minimizing the estimation error magnitude.

Summing up, the StaRTrEK algorithm pipeline is the following:

1. **Pre-processing**
  Z-score normalization of time profiles;
  non-uniform sampling regularization (if needed);
2. **Optimization**
  selection of the regularization parameter $\alpha$ using the L-curve method;
  computation of the parameter vector $\theta$ by solving (12) for each gene pair and the corresponding error *q*
  construction of matrices *H* and *Q*;
3. **Filtering**
  computation of matrices $H_{rnd}$ and $Q_{rnd}$ using randomized data;
  selection of the error threshold $q_{\text {max}}$ according to the Kolmogorov-Smirnov test;
  removal of half-life estimations in the matrix *H* with a corresponding estimation error (MSE) larger than the previously selected threshold $q_{\text {max}}$;
4. **Averaging**
  computation of the median of the half-life estimations for each row (gene) of the matrix *H* resulting from the previous step.

## Results and Discussion

### Performance evaluation on artificial data

In this section, we provide an *in silico* validation of the algorithm by generating simulated data to provide a preliminary assessment of its ability to recover true values on a variety of plausible situations, i.e., by considering its performance sensitivity to changes in (i) the number of available time samples, (ii) the amount of noise affecting the measurements and (iii) the number of genes involved into the estimation procedure. Artificial data were generated according to the following dynamic equation describing the rate of change of a given gene expression *x*:

16 $$\begin{aligned} \frac{dx(t)}{dt} = \gamma u(t) - kx(t), \end{aligned}$$

where *u*(*t*) is the transcription rate, $\gamma$ is a positive scaling factor, $k=ln(2)/hl$ is the degradation rate and *hl* is the gene half-life. To provide biological plausibility to the simulated data, we estimated the values and range of the various parameters used, from experimental data of measurements of transcripts abundance over a time-course and their turnover [14]. Specifically, we generated artificial time-course gene expression profiles across a simulation time interval of [0, 150] min, assuming a smooth time-varying promoter activity function *u*(*t*) having a sinusoidal shape, fixing $\gamma = 1$ and $x(0)=0$, and sampling half-lives from a realistic interval from 10 to 100 min [2, 14, 15] (Supplementary Data). In addition, we assumed that the signal was corrupted by a general measurement noise according to the following equation:

17 $$\begin{aligned} z(t) = x(t) + v(t), \end{aligned}$$

where *v*(*t*) is the noise term. For any time *t*, *v*(*t*) was drawn from a normal distribution with zero mean and standard deviation depending on *x*(*t*). Precisely, the standard deviation was taken as a percentage of the current state, i.e., $s.d.(v(t)) = C*x(t)$ with $C \in [0, 1]$.

The performance indices we considered were the Pearson correlation $\rho$ between the measured and estimated half-lives (a), the corresponding *p* value (b), and the number of genes (percentage) having a $FDR<0.05$ (c).

In order to test the algorithm performances based on the data availabilities, we applied the StaRTrEK estimation procedure to different artificial data scenarios in which we varied the number of the available time samples, or the amplitude of the measurement noise, or the number of half-lives to be estimated. Tables 1, 2 and 3 report the numerical results of the simulations described above. In particular, Table 1 reports the performance indices (a)–(c) obtained by decreasing the number of the available time samples *n* from 12 to 6, while keeping fixed the number of half-lives to be estimated ($m = 1000$) and the noise size ($C = 0.2$); Table 2 reports the performance indices (a)–(c) obtained by increasing the noise size *C* from 0.1 to 0.5, while keeping fixed the number of time samples ($n = 6$) and the number of half-lives to be estimated ($m = 1000$); Table 3 reports the performance indices (a)-(c) obtained by decreasing the number of half-lives to be estimated *m* from 1000 to 200, while keeping fixed the number of time samples ($n = 6$) and the noise size ($C = 0.2$).

As a general comment we note that the StaRTrEK algorithm reached very high performances in the simulated scenarios. Indeed, except for the case $C=0.5$ in Table 2, we obtained very high correlations in the range 0.7–0.9, and *p* values always much smaller than the statistical significance threshold (i.e., 0.05). It is worth noting that the proposed algorithm works extremely well also on short time-series (i.e., $n = 6$), which is the most important advantage of the algorithm. Moreover, an increasing in the noise size leads to a reduction in correlation (up to 0.11 for $C = 0.5$), whereas the reduction of the half-lives to be estimated slightly affects the algorithm performances.

**Table 1:** Performance indices on artificial data of half-lives and expression time-courses with different numbers of time samples (*n*)

	$\alpha _{opt}$	$q_{\text {max}}$	$\rho$	*pval*	$FDR<0.05$
$n=12$	10	3	0.89	$<10^{-309}$	$92.5\%$
$n=10$	10	3	0.89	$<10^{-309}$	$94.4\%$
$n=8$	10	4	0.89	$<10^{-309}$	$94.2\%$
$n=6$	10	2	0.86	$1.3\cdot 10^{-277}$	$95.2\%$

Number of gene expression profiles ($m = 1000$) and noise amplitude ($C=0.2$) are kept fixed. Legend—$\alpha _{opt}$: optimal value of the regularization parameter; $q_{\text {max}}$: error threshold expressed as percentile of the MSE distribution; $\rho$: Pearson correlation coefficient; *pval*: *p* value; *FDR*: false discovery rate. For each instance of the noise distribution considered, we found a negligible variability of the quality indices, therefore variance is not reported in the table

**Table 2:** Performance indices on artificial data of half-lives and expression time-courses with different noise amplitudes (*C*)

	$\alpha _{opt}$	$q_{\text {max}}$	$\rho$	*pval*	$FDR<0.05$
$C=0.1$	10	1	0.90	$<10^{-309}$	$99.6\%$
$C=0.2$	10	2	0.86	$1.3\cdot 10^{-277}$	$95.2\%$
$C=0.3$	10	2	0.86	$2.3\cdot 10^{-269}$	$91.1\%$
$C=0.4$	10	4	0.68	$8.9\cdot 10^{-123}$	$90.8\%$
$C=0.5$	10	10	0.11	$3.9\cdot 10^{-4}$	$90.8\%$

Numbers of gene expression profiles ($m = 1000$) and of time samples ($n=6$) are kept fixed. Legend—$\alpha _{opt}$: optimal value of the regularization parameter; $q_{\text {max}}$: error threshold expressed as percentile of the MSE distribution; $\rho$: Pearson correlation coefficient; *pval*: *p* value; *FDR*: false discovery rate. For each instance of the noise distribution considered, we found a negligible variability of the quality indices, therefore variance is not reported in the table

**Table 3:** Performance indices on artificial data of half-lives and expression time-courses with different numbers of half-lives to be estimated (*m*)

	$\alpha _{opt}$	$q_{\text {max}}$	$\rho$	*pval*	$FDR<0.05$
$m=1000$	10	2	0.86	$1.3\cdot 10^{-277}$	$95.2\%$
$m=800$	10	3	0.82	$3.0\cdot 10^{-180}$	$93.4\%$
$m=500$	10	3	0.78	$2.0\cdot 10^{-94}$	$91.0\%$
$m=200$	10	6	0.78	$3.5\cdot 10^{-38}$	$90.0\%$

Number of time samples ($n = 6$) and noise amplitude ($C=0.2$) are kept fixed. Legend—$\alpha _{opt}$: optimal value of the regularization parameter; $q_{\text {max}}$: error threshold expressed as percentile of the MSE distribution; $\rho$: Pearson correlation coefficient; *pval*: *p* value; *FDR*: false discovery rate. For each instance of the noise distribution considered, we found a negligible variability of the quality indices, therefore variance is not reported in the table

### Performance evaluation on experimental data

In this section, we present the results of the algorithm by considering three experimental datasets where both time-course and half-lives on a genome-wide scale has been measured (Supplementary Data). In particular, to show the performance of the proposed algorithm when dealing with a small number of samples, we focused on genome-wide yeast transcript half-lives and expression time-course data obtained in response to oxidative and DNA damage stress both collected by Shalem and co-workers [14], consisting of six time points for each gene and condition. Then, to show the performances over a long time-course (48 time points), we considered experimental data provided by [2, 15] taken during malaria intraerythrocytic developmental cycle (IDC). As performance indices we considered the Pearson correlation ($\rho$) between the measured and estimated half-lives, the corresponding *p*-value, and the number of genes (as percentage) having a $FDR<0.05$. For each dataset, we have also reported the selected values for the $\alpha$ parameter and $q_{\text {max}}$ threshold.

**Fig. 2:**
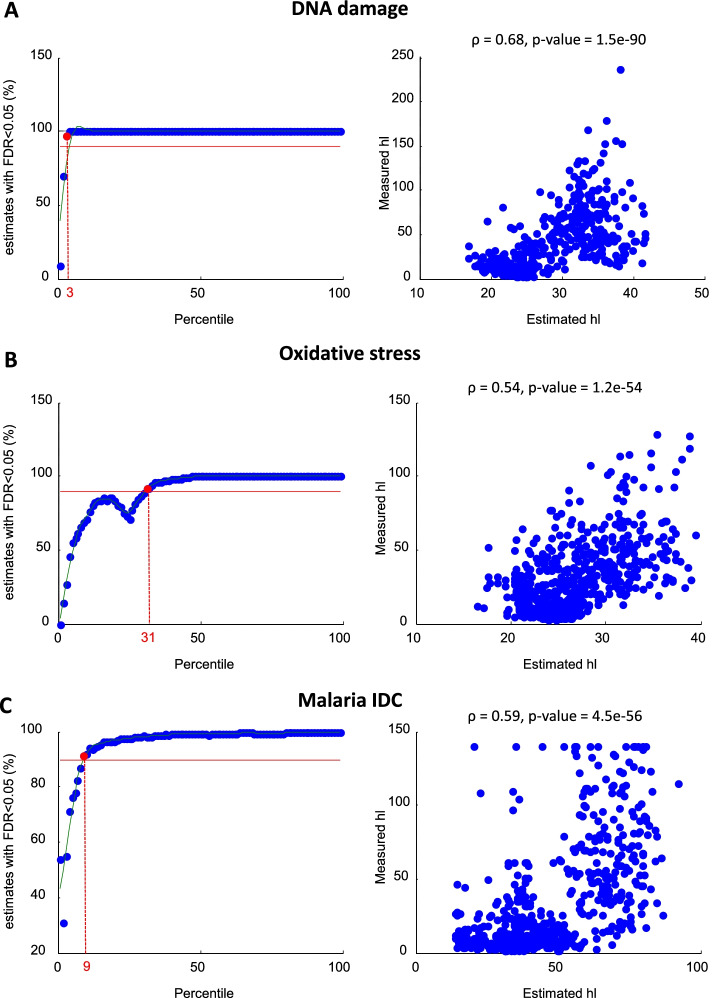
StaRTrEK algorithm validation using experimental data. StaRTrEK results obtained for yeast DNA damage (**A**), yeast oxidative stress (**B**), and malaria IDC (**C**) datasets. For each dataset, left panel shows the criterion for the selection of the error threshold $q_{\text {max}}$, whereas the right panel shows the scatterplot of RNA half-lives estimated by the StaRTrEK algorithm versus experimentally measured values

#### DNA damage dataset in yeast

This dataset includes genome-wide yeast transcript half-lives and expression time-course (6 time points at 0, 30, 60, 100, 140, 180 min) following exposure to methyl methanesulfonate (MMS), which induces DNA damage [14]. The majority of the responding genes showed a long enduring response with no relaxation. We selected genes with a fold ratio $>2$ and obtained 803 genes (Supplementary Data). The pre-processing step consisted only in the Z-score normalization since time sampling was almost uniform (30 or 40 min). The optimization step was performed using the optimal regularization parameter $\alpha = 7$, whereas, for the filtering phase, we selected the $q_{\text {max}}$ value corresponding to the $3^{th}$ percentile of the MSE distribution (Fig. 2A, left panel). The half-lives values resulting from StaRTrEK algorithm were in excellent agreement with the experimental measurements, reaching a Pearson correlation value of 0.68 and a *p* value = $1.5\cdot 10^{-90}$ (Table 4 and Fig. 2A, right panel).

**Table 4:** Summary of performance indices on experimental data. Legend—$\alpha _{opt}$: optimal value of the regularization parameter; $q_{\text {max}}$: error threshold expressed as percentile of the MSE distribution; $\rho$: Pearson correlation coefficient; *pval*: *p* value; *FDR*: false discovery rate

	$\alpha _{opt}$	$q_{\text {max}}$	$\rho$	*pval*	$FDR<0.05$
nDNA damage	7	3	0.68	$1.5\cdot 10^{-90}$	$98\%$
Oxidative stress	14	31	0.54	$1.2\cdot 10^{-54}$	$92\%$
Malaria IDC	15	9	0.59	$4.5\cdot 10^{-56}$	$91\%$

#### Oxidative stress dataset in yeast

This dataset includes genome-wide yeast transcript half-lives and expression time-course (6 time points at 0, 30, 60, 100, 140, 180 min) data following exposure to hydrogen peroxide ($H_2O_2$), which induces an oxidative stress [14]. The response kinetics is quite different from the DNA damage experiments since the majority of the responding genes showing a fast transient response. We selected genes with a fold ratio $>1.3$ and obtained 851 genes (Supplementary Data). As before, the pre-processing step consisted only in the Z-score normalization since time sampling was almost uniform (30 or 40 min). For this dataset, we selected the optimal regularization parameter $\alpha = 7$ and the $q_{\text {max}}$ value corresponding to the $31^{th}$ percentile of the MSE distribution (Fig. 2B, left panel), obtaining a Pearson correlation value of 0.54 and a *p* value = $1.2\cdot 10^{-54}$ (Table 4 and Fig. 2, left panel).

#### Malaria IDC dataset

This dataset includes genome-wide transcript half-lives and expression time-course (48 time points, one sample per hour) obtained during the malaria IDC [2, 15]. We selected the top 1000 genes in terms of the periodicity score defined in [2] (Supplementary Data). According to the algorithm pipeline, we initially performed only a Z-score normalization since the sampling times were uniform (1 h). The optimization step was performed using the optimal regularization parameter $\alpha = 15$, while, for the filtering phase, we selected the $q_{\text {max}}$ value corresponding to the $9^{th}$ percentile of the MSE distribution (Table 4 and Fig. 2C, left panel). Notably, the agreement between StaRTrEK estimations and the experimental measurements is excellent as witnessed by a Pearson correlation of about 0.6 and a *p* value euqal to $4.5\cdot 10^{-56}$ (Table 4 and Fig. 2C, right panel). It is worth noting that, although we have more points available than the previous cases, the correlation value and its significance does not improve. To explain this point, we note that the impact of half-life on the gene expression profile of a given RNA (as explained in detail in the review [13]) is apparent only when there are changes in the time-course; in fact, it is impossible to recover the half-life from a constant profile (i.e. at equilibrium) since such value is uniquely defined by the ratio of the half-life and the (constant) promoter activity. Therefore, even if more time points are available, only a small subset of them can effectively impact on the half-life estimation. Precisely, only those on the steep uphill and downhill faces of the gene expression time-profile determine the RNA half-life.

### Concluding remarks

The availability of genome-wide gene expression profiles have revolutionized life sciences at the molecular level. The analysis of the transcriptome goes far beyond DNA sequencing, since allows to put genes into action in the highly coordinated cell regulatory network. Recently, the discovery of a specific and extensive post-transcriptional regulation of gene expression level, has attracted many researchers to the study of transcripts kinetic, i.e., the behavior over time of a cell response. In fact, the transcript half-life value determines the shape of the time profile during changes, i.e., during transient responses like a switch-on / switch-off transition. In other words, RNA half-life is a very important measure of cell response to an internal or external changing environment. Usually, genome-wide gene expression time profiles experiments are composed of few samples, since the interest of the researcher is focused on the early, middle and late response, so that about 5 or 6 time points are usually collected, considering also the high costs of a genome-wide measurement. Transcripts half-lives can be obtained by a variety of methodologies like transcriptional inhibition or metabolic labeling, but the costs are high and the measurement procedure may impact the physiology of the cell under study, thus leading to possible artifactual results. Here, we showed how to recover half-lives directly from gene expression time courses using a computational model of RNA dynamics. The model here proposed is very simple but effective, it required only three parameters to be estimated and, in fact, we showed a significant agreement between estimated and measured half-lives using two experimental datasets collecting 6 time samples. We believe that our algorithm can be used as a fast valuable computational complement to time-course experimental studies by adding a relevant kinetic property with a strong biological interpretation.

Finally, we note that our method tends to underestimate half-life values. This observation actually needs an explanation or, at least, to suggest one. We did not observe this underestimation using artificial data, so we guess that it may have a biological reason rather than computational. To this aim, we note that the measured half-lives are obtained after transcriptional inhibition, whilst our algorithm makes use of the gene expression dataset where both transcription and degradation are present. It is well known that transcriptional inhibition has a large impact on the RNA half-life values since RNA half-life regulation is blocked and the experimental environment is far from a physiological status. By contrast, our computational analysis is based on more physiological data that refer to the specific biological process under study and, as such, it should be more reliable. Obviously, this claim needs experimental validation, but it is certainly reasonable. Finally, this observation also suggests the intriguing possibility that transcriptional inhibition impacts RNA half-lives by increasing their values.

## Supplementary Information

**Additional file 1.** Results of the StaRTrEK algorithm using experimental and artificial (simulated) data.

## Data Availability

The StaRTrEK methodology was implemented in Matlab and the source code can be made available upon request from the corresponding author. The data analyzed in this paper were taken from [2, 14, 15]. DNA damage and oxidative stress response datasets can be downloaded from: https://www.ncbi.nlm.nih.gov/geo/query/acc.cgi?acc=GSE12222 and Malaria datasets from: https://doi.org/10.1371/journal.pbio.0000005.sd004 and https://static-content.springer.com/esm/art
